# Molecular Modeling Studies of 11β-Hydroxysteroid Dehydrogenase Type 1 Inhibitors through Receptor-Based 3D-QSAR and Molecular Dynamics Simulations

**DOI:** 10.3390/molecules21091222

**Published:** 2016-09-19

**Authors:** Haiyan Qian, Jiongjiong Chen, Youlu Pan, Jianzhong Chen

**Affiliations:** 1 College of Pharmaceutical Sciences, Zhejiang University, Hangzhou 310058, Zhejiang, China; hyqian11119010@zju.edu.cn (H.Q.); 11319001@zju.edu.cn (Y.P.); 2 The Children’s Hospital, Zhejiang University School of Medicine, Hangzhou 310003, Zhejiang, China; xiaojiong@zju.edu.cn

**Keywords:** 11β-HSD1, 3D-QSAR, MESP, MD simulations, binding free energy

## Abstract

11β-Hydroxysteroid dehydrogenase type 1 (11β-HSD1) is a potential target for the treatment of numerous human disorders, such as diabetes, obesity, and metabolic syndrome. In this work, molecular modeling studies combining molecular docking, 3D-QSAR, MESP, MD simulations and free energy calculations were performed on pyridine amides and 1,2,4-triazolopyridines as 11β-HSD1 inhibitors to explore structure-activity relationships and structural requirement for the inhibitory activity. 3D-QSAR models, including CoMFA and CoMSIA, were developed from the conformations obtained by docking strategy. The derived pharmacophoric features were further supported by MESP and Mulliken charge analyses using density functional theory. In addition, MD simulations and free energy calculations were employed to determine the detailed binding process and to compare the binding modes of inhibitors with different bioactivities. The binding free energies calculated by MM/PBSA showed a good correlation with the experimental biological activities. Free energy analyses and per-residue energy decomposition indicated the van der Waals interaction would be the major driving force for the interactions between an inhibitor and 11β-HSD1. These unified results may provide that hydrogen bond interactions with Ser170 and Tyr183 are favorable for enhancing activity. Thr124, Ser170, Tyr177, Tyr183, Val227, and Val231 are the key amino acid residues in the binding pocket. The obtained results are expected to be valuable for the rational design of novel potent 11β-HSD1 inhibitors.

## 1. Introduction

Obesity, diabetes mellitus and other metabolic syndrome manifestations are primary causes of morbidity and mortality around the world [1]. The metabolic syndrome comprises an increased risk of type 2 diabetes and hyperlipidemia, which is related to a loss of insulin sensitivity in tissues including adipose tissue, muscle and liver [2,3,4]. One characteristic feature of visceral adipose tissue is its particular responsiveness to a steroid hormone glucocorticoid, which plays an essential role in regulating glucose and lipid homeostasis [5]. An excessive level of glucocorticoid may be associated with metabolic syndromes, like insulin resistance, central obesity, hyperglycemia, dyslipidemia, hypertension, and type 2 diabetes [6,7,8]. The effects of glucocorticoid depend not only on the circulating cortisol levels, but also on the intracellular production of cortisol through reduction of cortisone. The enzymes catalyzing the conversion between cortisone and cortisol are 11β-hydroxysteroid dehydrogenases (11β-HSDs). Among them, 11β-HSD1 is widely expressed in many glucocorticoid target tissues, notably in liver, adipose tissue, and muscle [5,9]. In these tissues, it acts as an NADPH-dependent reductase converting cortisone to the metabolic hormone cortisol [10], thereby locally amplifying glucocorticoid action. Sharing only 21% sequence identity with 11β-HSD1, 11β-HSD2 is an NAD^+^ dependent dehydrogenase that catalyzes the oxidization of cortisol to cortisone. This isozyme is mainly located in classical mineralocorticoid target tissues, such as salivary glands, kidney and colon, and its function is to protect the mineralocorticoid receptor from activation by cortisol [11,12]. Although 11β-HSD1 and 11β-HSD2 both catalyze the interconversion of glucocorticoids, they are functionally quite different. Inhibition of 11β-HSD1 may control the cortisol concentrations in liver and adipose without affecting the systemic circulation levels. For example, the level of active glucocorticoids was demonstrated to be increased in adipose tissue of transgenic mice with overexpressed 11β-HSD1. The phenotype of these mice developed visceral obesity, insulin resistance and reduced glucose tolerance [13]. These findings were in accordance with all aspects of the human metabolic syndrome, particularly without raising circulating glucocorticoid levels. Conversely, 11β-HSD1 knockout mice were unable to convert inert 11-dehydrocorticosterone to corticosterone in vivo. The cells in adipose tissue and liver couldn’t produce bioactive glucocorticoids, and the mice were resistant to diet-induced obesity and had increased insulin sensitivity [14]. These observations led to a drive to focus attention on the development of 11β-HSD1 inhibitors that were effective at constraining the activity of the enzyme in metabolic tissues. Therefore, 11β-HSD1 was proposed to be a therapeutic target for the treatment of metabolic syndromes and diabetes mellitus [15,16].

A number of small molecular inhibitors of 11β-HSD1 have been disclosed in the past few years [17,18,19], and a few of compounds have got into clinical trials studies. Incyte’s small molecule inhibitor INCB-13739 had completed phase I trials and was progressed into phase II trials in 2007. The phase II clinical trials data showed that INCB-13739 significantly improved insulin sensitivity in type 2 diabetic patients who failed in metformin treatment, and reduced the levels of triglyceride and cholesterol in hyperlipidemic patients [20,21]. UE-2343 from the University of Edinburgh (structure not disclosed) is being evaluated in clinical trials [22]. HPP851 from High Point Pharmaceuticals (structure not disclosed) is also being studied in a phase I/IIa clinical trial for the treatment of glaucoma [23].

As a useful technology and tool for drug design, computer-aided drug design methods have been applied to discover and design new 11β-HSD1 inhibitors [24,25,26,27]. However, few molecular dynamics simulations were reported to investigate the detailed interaction of 11β-HSD1 and inhibitors, which would be useful molecular information for design of novel 11β-HSD1’s inhibitors. Herein, in order to provide helpful clues about the rational modification of molecules for the design of more potent 11β-HSD1 inhibitors, molecular modeling studies combining three-dimensional structure-activity relationship (3D-QSAR) analysis, molecular docking, molecular electrostatic profile (MESP) analysis, molecular dynamics (MD) simulations, molecular mechanics Poisson-Boltzmann surface area (MM/PBSA) and molecular mechanics Generalized-Born surface area (MM/GBSA) methods were conducted to investigate the detailed binding modes between 11β-HSD1 and its inhibitors and also to find the key structural features affecting the inhibitory activities. To obtain the rational conformation for developing 3D-QSAR models, we applied the docking-based conformation selection strategy. Moreover, the specific binding modes were analyzed on the basis of the results from MD simulations and free energy calculations for three compounds with diverse structures and different activities bound to 11β-HSD1. The obtained results would not only help to better understand receptor-ligand interactions but also provide some insights into the further structural modification for developing novel potent 11β-HSD1 inhibitors.

## 2. Calculation Methods

### 2.1. Data Set and Biological Activity

A total of 40 11β-HSD1 inhibitors, including pyridine amides and 1,2,4-triazolopyridines analogs, were collected from literatures reported by the same research group [28,29] to be used as a data set for molecular modeling. The molecular structures as well as biological activities are listed in Table S1 in the Supplementary Materials. The IC_50_ values were converted into the corresponding pIC_50_ values and were used as dependent variables for subsequent 3D-QSAR analyses. The whole data set was divided into a training set of 31 compounds for 3D-QSAR model generation and a test set of 9 compounds for model validation, respectively, by considering both distribution of biological data and structural diversity. As shown in Figure S1, compounds in either training or test set should have their bioactivities spanning more than four orders of magnitude and be well distributed in each activity scale. 

### 2.2. Molecular Modeling and Data Set Alignment

The co-crystal structure (PDB ID: 3CH6 [28]) of 11β-HSD1 with compound **1** was retrieved from RCSB Protein Data Bank (http://www.rcsb.org/pdb/). 3D structures of all other compounds in both training and test sets were generated by modifying corresponding functional groups of **1** with the SKETCH module of Sybyl-X1.3 molecular modeling software package [30]. Subsequently, the constructed structures were energy-minimized using Tripos force field with a distance-dependent dielectric function and Powell conjugate gradient method with a convergence criterion of 0.001 kcal/(mol·Å). The partial atomic charges were assigned on the basis of the Gasteiger-Hückel formalism. Given the diversity of the data set containing different backbones, molecular docking was applied to align the data set compounds.

To explore the binding conformation of the above compound in the active site of 11β-HSD1, flexible docking simulations were performed using the Surflex-Dock program in Sybyl-X1.3 [31]. The primary conformation of compound **1** engaged in the co-crystal structure was used as a starting position to define the potential binding site. Each compound was then docked into the idealized active site with a “whole” molecular alignment algorism [32] and the putative poses were ranked using the Hammerhead scoring function [33]. Herein, two parameters determining the extent and volume of binding site were kept at default settings (threshold = 0.50 and bloat = 0 Å). During the docking process, Consensus Score (CScore) calculations were performed and all other adjustable parameters were kept at their default values. Twenty docked poses with high calculated interaction scores were finally saved for each inhibitor. The desired docked conformation was chosen from the output poses based on two criteria [34], the conformation with the highest docking score and the conformational orientation similar to the ligand in the co-crystal structure.

### 2.3. 3D-QSAR Models Generation

Both comparative molecular field analysis (CoMFA) and comparative molecular similarity indices analysis (CoMSIA) methods were applied for the development of 3D-QSAR models using the QSAR module of Sybyl-X1.3. To generate CoMFA model, steric and electrostatic fields were calculated at each point of regularly spaced grids of 2.0 Å using an *sp*^3^ hybridized carbon atom probe with a charge of +1.0 and van der Waals radius of 1.53 Å. The cut-off value was set to the default value of 30.0 kcal/mol. For CoMSIA analyses, by using a common probe with a charge of +1.0, five similarity indices consisting of steric (S), electrostatic (E), hydrophobic (H), H-bond donor (D), and H-bond acceptor (A) fields were calculated for each lattice with a grid of 2 Å. A Gaussian method was used to evaluate the mutual distance between the probe atom and each molecule atom in CoMSIA model generation. The attenuation factor was set to the default value of 0.3.

Partial least squares (PLS) [35,36] method was used for the 3D-QSAR investigation, in which field descriptors were employed as the independent variables and inhibitory activity (pIC_50_) served as target variables. A minimum column filter value (σ) of 2.0 kcal/mol was set to reduce noise and speed up the analysis. Leave-one-out (LOO) algorithm was adopted in the cross-validation analysis to obtain an optimal number of components (ONC), the lowest standard error of prediction and cross-validated correlation coefficient (*q*^2^). Then, the obtained ONC was used to calculate the non-cross-validated correlation coefficient (*r*^2^) of the produced PLS model. In addition, the statistical significance of the generated models was described by the standard error of estimate (SEE) and *F* probability value (*F* value). The predictive capability of the 3D-QSAR models was further evaluated with the test set of molecules. The predictive correlation (*r*^2^_pred_) based on the test set molecules was computed using Equation (1) below:

(1) $${r^{2}}_{\mathrm{pred}}=1-\frac{\mathrm{PRESS}}{\mathrm{SD}}$$

where PRESS is the sum of squared deviation between predicted and actual activity values for each molecule in the test set, and SD is the sum of the squared deviations between the biological activities of the test set and mean activities of the training set molecules.

### 2.4. Molecular Electrostatic Potential Calculation

Molecular electrostatic potential (MESP) is a useful feature in understanding the molecular electronic structure, chemical reactivity of the molecule, and structure-activity relationship studies [37]. In the current study, MESP was applied to inspect the electrostatic effects of compounds binding to 11β-HSD1 in combination with 3D-QSAR analyses. In the meantime, the highest occupied molecular orbitals (HOMO), the lowest unoccupied molecular orbitals (LUMO), and dipole moments of compounds **1**, **14**, **32**, and **39** were calculated to explain bioactivities and molecular properties using *ab initio* method. The docking-predicted conformation of each compound was used as an input conformer for density functional theory (DFT) calculations using Gaussian 09 program package [38]. Complete geometry optimization was carried out with the DFT theory using B3LYP method based on the basis set 6-31g(d,p) level [39,40]. In order to simulate the physiological conditions, all energy calculations were carried out with a CPCM model to mimic the aqueous environment [41]. MESP and Mulliken atomic charges of each molecule were analyzed using GaussView [42]. In detail, HOMO and LUMO orbitals were generated and visualized at an isodensity value of 0.02. The electrostatic potentials were scanned over the entire surface of the molecule to measure the charge distribution. 3D isosurface of MESP was plotted onto a surface of constant electrostatic electron density (0.0004 e/au^3^). The overall molecular size and the distribution of positive or negative electrostatic potential were indicated by color coded isosurface values. The most negative and positive electrostatic potentials are colored deep red and blue, respectively, and the intermediated shades colored with cyan, yellow, and green indicate the moderate range of reactivity.

### 2.5. Molecular Dynamics Simulations

The MD simulations were performed to investigate different interaction modes of ligands with various bioactivities binding to 11β-HSD1 using the AMBER12 software package [43]. The docking-simulated structural models of complexes 11β-HSD1-**1**, 11β-HSD1-**11**, and 11β-HSD1-**14** were used as initial structures for MD computations, respectively. The force field parameters for each inhibitor were generated by the general AMBER force field (GAFF) using ANTECHAMBER program [44], and the restrained electrostatic potential (RESP) charges [45] were used to assign the partial atomic charges for the ligand atoms by fitting the electrostatic potentials calculated by Gaussian 09 at the HF/6-31G* level of theory [38]. The standard *ff03.r1* force field was applied to obtain force field parameters for protein and water molecules [46]. All missing hydrogen atoms of the protein were added using the LEaP program [47]. Considering protonation states of ionizable amino acid residues at a neutral pH value, all models were neutralized by adding appropriate counterions (Na^+^ or Cl^−^ ions). Each complex was solvated in a truncated octahedron box of TIP3P water molecules [48] with a margin distance of 9 Å to get corresponding water solvation system. Prior to MD simulations, each system was subjected to three steps of energy minimization conducted by the SANDER module in AMBER12 to prevent any steric conflict occurred during system setup. Firstly, all counterions and water molecules were optimized, while both protein and ligand were kept frozen with a constraint potential of 500 kcal/(mol∙Å^2^). Secondly, residue side chains of protein were relaxed while backbone heavy atoms were restrained with a constraint force of 5 kcal/(mol∙Å^2^). Finally, the whole system was optimized without any restraint. In each step, structural optimization was implemented using 2500 steps of steepest descent followed by 5000 steps of conjugate gradient method.

After energy minimizations, each system was gradually heated in the NVT ensemble from 0 to 300 K over a period of 100 ps with a force constant of 10 kcal/(mol∙Å^2^) on the protein-ligand complex. Subsequently, the whole system was subjected to seven rounds of equilibrations in each time period of 1 ns at 300 K with a carefully decreasing restraint weights of 5, 3, 1, 0.5, 0.3, 0.1, and 0 kcal/(mol·Å^2^), respectively, without restriction on the solvation environment. Finally, 50 ns MD productions were run for each system in the NPT ensemble at 300 K with 1.0 atm pressure. During MD simulations, periodic boundary conditions were applied to avoid edge effects in all calculations. The particle mesh Ewald (PME) method was utilized to deal with the long-rang Coulombic interactions [49,50] and the cutoff distance was set at 10 Å for the long-range van der Waals (vdW) energy term. The SHAKE algorithm was employed on all atoms covalently bond to hydrogen atoms along with an integration time step of 2 fs [51]. Coordinate trajectories were recorded with a collision frequency of 1.0 ps^−1^ throughout the equilibration runs and with a collision frequency of 2.0 ps^−1^ throughout the production runs.

### 2.6. Binding Free Energy Calculations and Decomposition Analysis

For each complex, 800 snapshots were extracted from the last 4 ns MD trajectories at 5 ps intervals to calculate the binding free energy using the parallelized python script *MMPBSA.py.MPI* in AMBER12 [52]. For each snapshot, the binding free energy was calculated of each molecular species (complex, protein, and ligand):

Δ*G*_bind_ = Δ*G*_complex_ − (Δ*G*_protein_ + Δ*G*_ligand_) = Δ*E*_MM_ + Δ*G*_sol_ − *T*Δ*S* (2)

where Δ*E*_MM_ is the molecule mechanics energy, which was calculated by the electrostatic and vdW interactions and the internal energy term (bond, angle, and dihedral energies):

Δ*E*_MM_ = Δ*E*_ele_ + Δ*E*_vdW_ + Δ*E*_internal_ (3)

Since there is no covalent bond formed between the ligand and receptor, Δ*E*_internal_ is omitted in the MD calculation.

Δ*G*_sol_ is the solvation free energy, which was composed of the polar and the nonpolar contributions:

Δ*G*_sol_ = Δ*G*_pol_ + Δ*G*_nonpol_ (4)

where the polar part Δ*G*_pol_ could be obtained by solving the Poisson-Boltzmann (PB) equation for MM/PBSA method [53] or the Generalized-Born (GB) equation for MM/GBSA method [54]. Whereas, Δ*G*_nonpol_ was estimated by:

Δ*G*_nonpol_ = γSASA + β (5)

The solvent accessible surface area (SASA, Å^2^) was computed with a probe radius of 1.4 Å using the linear combination of pairwise overlaps (LCPO) method [55]. In this work, the surface tension parameter (γ) and offset (β), being a constant, were set to 0.005420 kcal/(mol∙Å^2^) and −1.008000 kcal/mol for PB calculations and 0.0072 kcal/(mol∙Å^2^) and 0 kcal/mol for GB calculations, respectively [53].

The entropy (*T*Δ*S*) was obtained from changes in the translational, rotational and vibrational degrees of freedom. It can be determined from the following equation:

*T*Δ*S* = *T*(Δ*S*_translatio*n*_ + Δ*S*_rotational_ +Δ*S*_vibrational_) (6)

in which classical statistical thermodynamics can be used to calculate the first two terms, and the contribution from the vibrational degrees of freedom calculated by normal-mode analyses using the NMODE module in AMBER12 [45]. Prior to the normal-mode calculations, structural minimizations and normal-mode analyses for the complex, protein, and ligand were carried out with a distance-dependent dielectric function (ε = 4*r*_ij_) to mimic the impact of solvent. In consideration of the high computational demand of this approach, only 40 snapshots taken from last 4 ns MD trajectories at 100 ps intervals were utilized to estimate the entropic contribution.

In order to investigate detailed binding modes between the protein and its inhibitors and to find key residues affecting the inhibitory activity, binding free energies were decomposed to each residue′s contribution to ligand using MM/GBSA method. There were only molecular mechanic energies and solvation energies but not for entropies taken into consideration in this decomposition. The binding interaction of per inhibitor-residue includes four terms, van der Waals contribution (Δ*E*_vdW_), electrostatic contribution (Δ*E*_ele_), polar solvation contribution (Δ*G*_pol_), and nonpolar solvation contribution (Δ*G*_nonpol_). The vdW and electrostatic interactions between inhibitor and per-residue of 11β-HSD1 were calculated using the SANDER program of AMBER12. The polar contributions of the solvation free energy were computed by the GB model. The nonpolar contributions of the desolvation free energy were determined with SASA dependent terms. All energy decomposition analyses were performed on the basis of the same snapshots which were used in previous calculations.

## 3. Results and Discussion

### 3.1. Docking Analysis

In this study, flexible docking calculations were performed on inhibitors of 11β-HSD1 to predict their potential binding conformations using the Surflex-Dock procedure. As shown in Figure S2a in the Supplementary Materials, the obtained docking pose of **1** was similar to its experimental conformation in the co-crystal structure with a root mean-square deviation (RMSD) of 0.39 Å, indicating the high reliability of Surflex-Dock in reproducing the experimental binding mode of 11β-HSD1 inhibitors. Figure S2b displays all 40 inhibitors docked into the binding pocket of 11β-HSD1. Although the spatial positions of the scaffolds were not kept in the alignment, all molecules share a similar binding mode.

The detailed binding mode of **1** in 11β-HSD1 is depicted in Figure S2c. Its amide carbonyl oxygen forms two hydrogen bonds (black dashes) with the side chain hydroxyl groups of residues Ser170 and Tyr183 with distances of 2.67 Å and 2.70 Å, respectively. The 3-fluoro-4-methylphenyl moiety is embedded in a hydrophobic cavity composed of Leu126, Tyr177, Met179, Val180, and Val231. It was further observed that both the 3-fluoro-4-methylphenyl moiety and the central pyridine core of **1** may have strong parallel π-π interactions with the side chain phenyl ring of Tyr177. Moreover, the central pyridine core is also parallel to the backbone of Gly216 and Leu217 with van der Waals interaction. The 3,3-dimethylpiperidinyl moiety is surrounded by the hydrophobic side chains of Ile121, Thr124, Tyr183, Ala223, Ala226, and Val227.

**Figure 1:**
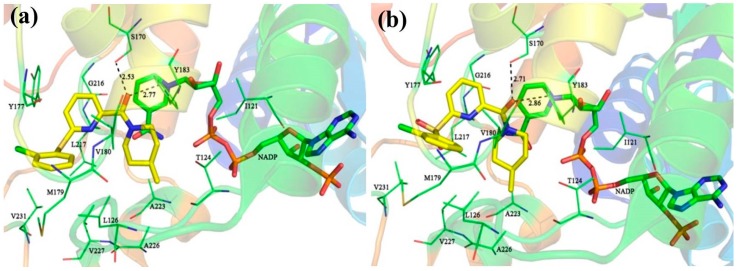
The detailed binding modes of complexes 11β-HSD1-**11** (**a**) and 11β-HSD1-**14** (**b**).

For comparison, compounds **5**, **11**, **14**, and **39** were selected for detailed analyses of their binding mechanism on 11β-HSD1. The binding modes of these four compounds combined with the main residues of 11β-HSD1 are displayed in Figure 1 and Figure S3 in the Supplementary Materials. Particularly, the amide carbonyl oxygens of **5**, **11**, **14** and N1 and N2 atoms of **39** form hydrogen bonds with the side chain hydroxyl groups of residues Ser170 and Tyr183. Thus, these two residues may provide crucial hydrophilic interactions with an inhibitor binding to 11β-HSD1. In addition, as compared to the highly active compound **1**, compound **5** contains a less hydrophobic 2-chlorophenyl moiety and a 4-methylpiperidinyl group resulting in a smaller hydrophobic space-filling in the binding pocket (Figure S3a). This result would explain why compound **5** has lower bioactivity than **1**. As for compound **11** or **14** (Figure 1a,b), a relatively weak T-shaped π-π interaction is formed between the 2-chlorophenyl group and the side chain phenyl ring of Tyr177 due to a single heteroatom linker (S or SO_2_) between the central pyridine core and the 2-chlorophenyl moiety. Moreover, compound **14** with a strong electron withdrawing linkage (SO_2_) is detrimental to 11β-HSD1 potency. On the other hand, inactive compound **39** has a different binding mode in comparison to **1**. Typically, the 2-methyl-4-chlorophenyl substituent of **39** approaches to a solvent exposed area of the binding site and is close to the hydrophobic residue Tyr177 with a weak T-shaped π-π interaction. The *N*-acetylpiperidin-4-yl moiety is oriented to another side of the binding pocket, reducing the hydrophobic interactions with the side chain of Ile121 (Figure S3b). Moreover, its polar acetyl substituent is also disfavored for interacting with 11β-HSD1. Due to the fact that the pocket is limited by residues Ile121, Thr124, and Tyr183, a modest steric hindrance could be beneficial for a ligand binding to 11β-HSD1. According to various bioactivities of these compounds, it may be hypothesized that favored hydrophobic and hydrophilic interactions would be beneficial to improve the inhibitory potency of a ligand.

### 3.2. Statistical Results of the CoMFA and CoMSIA Models

The statistical parameters of the generated CoMFA and CoMSIA models are summarized in Table 1. The best CoMFA model gave a cross-validated correlation coefficient *q*^2^ of 0.656 with an ONC of 5, a non-cross-validated *r*^2^ of 0.964, SEE of 0.286, and *F* value of 132.964. The corresponding field contributions of parameters were 58.6% of steric fields and 41.4% of electrostatic fields, indicating a greater influence of steric fields on the bioactivity. Such a prediction would be consistent with the results from the MD simulations discussed later. 

The CoMSIA model was generated based on five fields, steric field (S), electrostatic field (E), hydrophobic field (H), H-bond donor (D), and H-bond acceptor (A), giving a cross-validated *q*^2^ of 0.523 and a non-cross-validated *r*^2^ of 0.936 with an ONC of 6. The corresponding field contributions were 10.4% (S), 26.4% (E), 27.1% (H), 18.0% (D), and 18.1% (A), respectively. The electrostatic and hydrophobic contributions shared large parts for the inhibitory activity for 11β-HSD1 in the generated CoMSIA model. It was noticed that steric contribution in the CoMSIA model was much lower than that in the CoMFA model. As we know, the CoMFA method is restricted to electrostatic fields with Lennard-Jones and Coulomb potentials. This may introduce errors in scaling, alignment sensitivity, and interpretation of contours. However, the CoMSIA method has been developed to make usage of hydrophobic fields in addition to the electrostatic fields. It can improve these inherent deficiencies arising from the CoMFA method [56]. Accordingly, there is 27.1% of hydrophobic field in addition to steric field (10.4%) in the generated CoMSIA model. In our previous docking study, it has been recognized that the hydrogen bond and hydrophobic interaction would play significant roles in the protein-ligand binding affinity. It also proves that the hydrophobic properties are important in the design of 11β-HSD1 inhibitors. The predictive capabilities of the generated CoMFA and CoMSIA models were further evaluated using the test set of compounds excluded from the construction of 3D-QSAR models. The experimental and predicted pIC_50_ values and residuals defined as the experimental minus the predicted pIC_50_ values are summarized in Table S2 in the Supplementary Materials. It was found that the predicted values of molecules in both training and test sets were close to the actual pIC_50_ values without deviation of more than one logarithmic unit. The correlations between the experimental and model-predicted bioactivities of all compounds are shown in Figure S4 in the Supplementary Materials. The predictive correlation coefficients *r*^2^_pred_ of the CoMFA and CoMSIA models were 0.938 and 0.923, respectively. The high *r*^2^_pred_ values indicate a good predictive ability of our generated 3D-QSAR models.

### 3.3. Interpretation of the CoMFA and CoMSIA Models Based on Receptor

**Figure 2:**
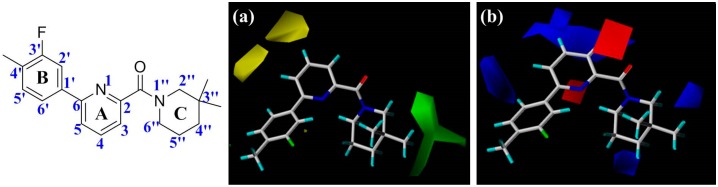
The contour maps of CoMFA model based on compound **1** (color figure online). (**a**) Steric field: green and yellow contours indicate regions where bulky groups increase and decrease activity. (**b**) Electrostatic field: red and blue contours indicate regions where negative charge increase and decrease activity.

**Figure 3:**
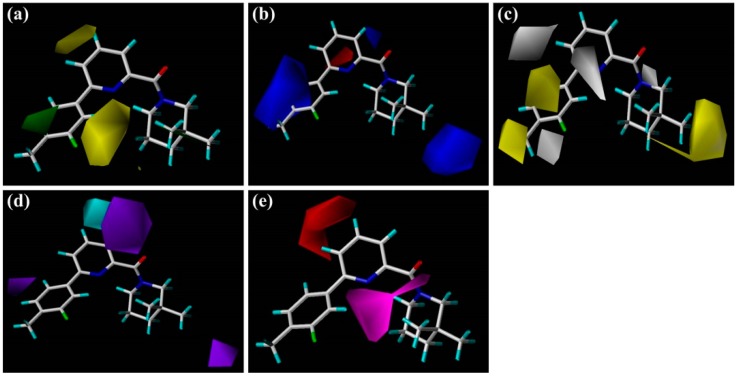
The contour maps of CoMSIA model based on compound **1**. (**a**) Steric field: green and yellow contours indicate regions where bulky groups increase and decrease activity; (**b**) Electrostatic field: red and blue contours indicate regions where negative charge increase and decrease activity; (**c**) Hydrophobic field: yellow and gray contours indicate favorable and unfavorable hydrophobic groups; (**d**) H-bond donor field: cyan and purple contours indicate favorable and unfavorable H-bond donor groups; (**e**) H-bond acceptor field: magenta and red contours indicate favorable and unfavorable H-bond acceptor groups.

The CoMFA and CoMSIA results were graphically described by the field contribution maps using the STDEV*COEFF field type. Figure 2 and Figure 3 display the contour maps derived from the generated CoMFA and CoMSIA models, respectively. Compound **1** was labeled on the map as a template for visualization to explore the structure-activity relationships of these 11β-HSD1 inhibitors. The contour analyses have been performed by dividing the total molecular area into three subdivisions (Figure 2), including the central pyridine or TZP core (A-ring), the distal aromatic ring (B-ring), and the aliphatic moiety (C-ring).

In Figure 2a, the contour map of the steric field of CoMFA model, a large green contour is close to 3″-position of C-ring, indicating that this area would prefer a bulky substituent rather than a small branch. According to the docking simulations, C-ring was pointed toward a hydrophobic pocket composed of residues Ile121, Thr124, Tyr183, Ala223, Ala226, and Val227. Moreover, this region could also be demonstrated in the hydrophobic contour map of the CoMSIA model with a large yellow polyhedron at the same position (Figure 3c), indicating that a hydrophobic group is favorable for bioactivity. This observation in either CoMFA or CoMSIA model is consistent with the experimental data that compound **2** with a 3″,3″-dimethyl group is more active than **5** with a smaller 4′-methyl substituent. Compounds **19**–**23** with diverse substituents on C-ring reflected the similar trend of bioactivity. Especially, compound **22** showed more than 800-fold higher bioactivity against 11β-HSD1 in comparison with **19**. On the other hand, there are two yellow moderate polyhedrons a little far away from the 4- and 5-positions of A-ring in the CoMFA model, indicating that bulky substituents in these positions may result in a decreased activity. Actually, our docking simulations indicated that the 4- or 5-position of **1**′s A-ring would point to a limited space composed of residues Leu171, Gly216, Leu217, and Met233. Therefore, bulky substituents in these places would be disadvantageous to the biological activities.

The electrostatic contour map of CoMFA model can be seen clearly from the Figure 2b. Since compounds **16** and **20**–**22**, which possess a nitrogen atom at the 4-position of A-ring, exhibited potent inhibitory activities, a small blue contour area is obtained near this site. Moreover, compound **30** with an electron-donating NH group among the ‘three-atom’ sulfonamide linker, which could be superimposed on the 4-position of **1**′s A-ring, has higher bioactivity than **29** with a ‘two-atom’ sulfone linker in the corresponding position. Another large blue contour is found around the 3- and 4-positions of A-ring. This contour could be used to explain why compound **18** with its nitrogen atom in pyridine ring is less potent than **15**. Besides, the blue contour near 6′-position of B-ring indicates that the considerable activity differences with an order of **9** > **15** > **10** could be due to the electronegativity of the corresponding linkers −SCH_2_−, −SO_2_CH_2_−, and −OCH_2_−. While two small blue polyhedrons at the 3″- and 4″-positions of C-ring reveal that an electropositive group at these positions would be beneficial for the binding affinity, like compounds **16** vs. **24**, **34** vs. **37**, and **35** vs. **36**. Conversely, a red region appears around the carbonyl oxygen of amide or the nitrogen atom at the 1-position of the TZP core, highlighting that an electronegative group is preferable at this position. This is in agreement with the docking results in which the carbonyl oxygen atom of highly active compound **1** accepts hydrogen bonds from the side chain hydroxyl groups of Ser170 and Tyr183. Another red region around the 1-position of the A-ring represents that electronegative group is favorable for enhancing bioactivity.

The steric and electrostatic contour maps of the CoMSIA model are depicted in Figure 3a,b, respectively. These contour maps were found to be nearly identical to the corresponding CoMFA contours except some minor differences. As shown in Figure 3a, there is a small green contour beside the 5′-position of **1**′s B-ring, suggesting that introduction of a hydrophobic substituent at this region would be beneficial to improve the inhibitory activity of a ligand. For instance, the activities of compounds **9** and **10** are higher than those of **11** and **12**. As compared with linker atoms between A-ring and B-ring in **11** (−S−) and **12** (−O−), the longer linkers of **9** (−SCH_2_−) and **10** (−OCH_2_−) may extend their phenyl ring B to the 5′-position of **1**′s B-ring, making **9** and **10** more similar to **1** in shape. In fact, the B-ring of **1** was positioned at the edge of the binding pocket of 11β-HSD1 and extended to the solvent in the docking-simulated structural model of complex 11β-HSD1-**1** (Figure S2c), thus it would allow a bulky substituent. In comparison, compound **11** or **12** has its aromatic ring B pointing to the medium-sized yellow contour near the 2′-position of **1**′s B-ring, suggesting that a steric substituent at this position degrades the biological activity of the molecule. Furthermore, compound **6** with 2′-NO_2_ or **8** with 2′-phenyl also showed slightly lower bioactivity than **1**. As for the electrostatic field (Figure 3b), three blue contours and one red contour are presented in similar sites like those in the CoMFA electrostatic map (Figure 2b). Since these contours were discussed above in the CoMFA model, our following discussion will focus on the CoMSIA hydrophobic contours as well as H-bond donor and acceptor contours.

As illustrated in the CoMSIA hydrophobic contour map (Figure 3c), the most parts of B-ring and 3″-position of C-ring are surrounded by yellow contours, suggesting that hydrophobic features at these positions are crucial for the inhibitory activity. Above docking results also disclosed that the 4′-substituted group of B-ring may interact with the hydrophobic residues Met179 and Val231. For example, compound **3** with 4′-Cl or **4** with 4′-OCF_3_ held high potency for 11β-HSD1 but showed slightly lower activity than compound **1** with 3′-F-4′-CH_3_. Meanwhile, compounds **25**, **30**, **32**, and **33**, bearing a suitable linker between A-ring and B-ring, made them be similar to **1** in molecular shape and exhibited higher activities than analog **28** with an oxygen linker. Furthermore, the 3″-position of C-ring would likely to occupy a sub-hydrophobic pocket composed of hydrophobic residues, like Ile121, Leu126, Val180, Tyr183, Ala223, and Ala226. Together, with the green contours in steric field map discussed above, it could be inferred that bulky and hydrophobic groups at these positions would enhance the inhibitory activity. On the other hand, two gray contours near the 1-position of A-ring and 1″-position of C-ring are congruent with the results obtained from the generated CoMFA model, showing that an electronegative atom would be desirable for an inhibitor. Another two gray areas appear at the 5-positon of A-ring and 3′-positon of B-ring, indicating that modifications of these parts with some hydrophilic substitutions could increase the bioactivity.

Figure 3d,e depict the graphical interpretation of the H-bond donor and H-bond acceptor features in the CoMSIA model, respectively. In Figure 3d, a cyan polyhedron is presented near the 4-position of **1**′s A-ring, suggesting that an H-bond donor would be favored to interact with the highly electronegative center. In fact, the NH group of sulfonamide linker in compound **30** was found to be superimposed on the 4-position of **1**′s A-ring, so it showed much higher potency than **29** with a sulfone linker. On the other hand, compound **31** has its NH group in sulfonamide linker pointing to the purple region presented around the 3-position of **1**′s A-ring. A piece of small purple contour near the 6′-position of B-ring may explain the lower activity of compound **13** with an NH group than **5**. Another small purple contour little far away from the 3″-position of C-ring is accordance with the decreased activity of compound **24** in comparison with **23**. As for H-bond acceptor contour maps (Figure 3e), a large magenta contour area covers the scaffold of compound **1** from the carbonyl oxygen of amide or N1-positon of the TZP core to the 2′-position of B-ring, indicating that groups with a hydrogen bond acceptor would be favored at the corresponding positions. Our docking simulations have also shown that the carbonyl oxygen atom or the nitrogen atoms of the TZP core form key hydrogen bonds with the side chain hydroxyl groups of Ser170 and Tyr183, which were considered as significant hydrogen bond interactions in the binding mode. This could be verified by the satisfactory potency of compound **6** bearing a nitro group at the 2′-position of B-ring. On the other hand, the medium-sized red contour beside the 4-position of A-ring reveals that an H-bond acceptor group there would be disadvantageous to the inhibitory activity.

### 3.4. MESP, HOMO-LUMO, and Mulliken Atomic Charges Analyses

To understand surface electronic properties of 11β-HSD1 inhibitors and to comprehend the pharmacophoric features required for binding, two highly active compounds (**1** and **32**), and two low activity compounds (**14** and **39**) selected from the docking study were analyzed based on MESPs, HOMO-LUMO parameters and dipole moments. In addition, Mulliken population analyses of studied inhibitors were also calculated to get more detailed insights at the electronic level.

**Figure 4:**
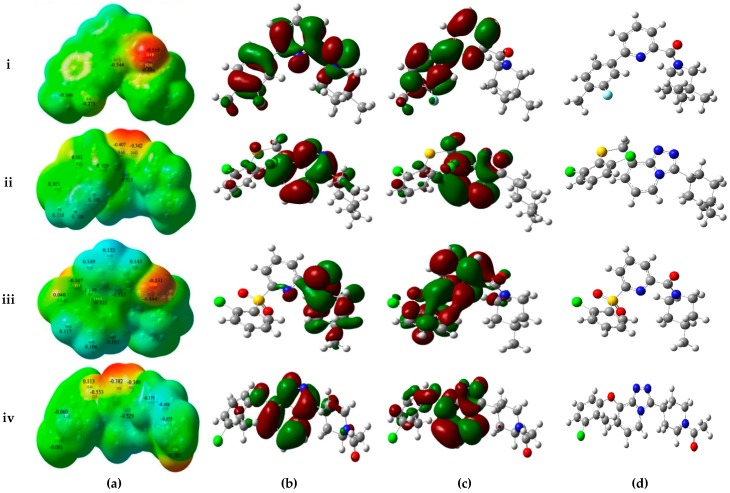
(**a**) MESP superimposed onto a surface of constant density and Mulliken atomic charges mapped onto the active compounds (**1** and **32**) and low active compounds (**14** and **39**) (i, ii, iii, and iv); (**b**) HOMOs; (**c**) LUMOs orbitals with B3LYP/6-31g(d,p); (**d**) the structures of the active compounds (**1** and **32**) and low active compounds (**14** and **39**). The variation in MESP from positive to negative is shown in red to blue scale.

As shown in Figure 4a, MESPs plotted on these four compounds reveal that the amide carbonyl oxygen atoms or the nitrogen atoms of the TZP core represent most electronegative potential regions (deep red color) favored for electrophilic attack. These results are in accordance with the medium-sized red contour in CoMFA model and purple and magenta contours in the CoMSIA model. Besides, previous docking results have also indicated the involvement of these areas in the important H-bond interactions with the key residues Ser170 and Tyr183. On the other hand, an apparent red region was observed near the electron-attracting oxygen atoms O1 and O44 of the sulfone linker in the low active compound **14** with an average Mulliken charge of −0.515, which does not fit with the blue contour around the 6′-position of B-ring in the electrostatic maps of 3D-QSAR models. Similarly, compound **39** shows strong electronegative potentials near the linker oxygen atom O5 with the Mulliken charge of −0.553, which is in conflict with the red contour in the H-bond acceptor contour of CoMSIA model. In addition, the carbonyl oxygen O20 of **39** exhibits the strongest electronegative potential (Mulliken charge: −0.507), which is also not in accordance with the large blue contour in the CoMSIA model. Thus, MESPs plotted over the low active compounds **14** and **39** indicated that their molecular electronic properties do not fit well with the required electrostatic features predicted by above generated 3D-QSAR models. 

The frontier orbitals HOMO and LUMO, which are quantum chemical descriptors, were calculated for these four molecules. The HOMO energy is closely related to reactivity to electrophilic attack while LUMO energy is closely related to reactivity to nucleophilic attack. Thus, the eigenvalues of HOMO and LUMO and their energy gap reflect the biological activity of the molecule. Usually, the decrease in the HOMO and LUMO energy gap explains the eventual charge transfer interaction taking place within the molecule under the influence of an external electric field. As shown in Figure 4b,c, the HOMO and LUMO sites were plotted onto the molecular surfaces of compounds **1**, **32**, **14**, and **39**, respectively. The distributions of HOMO and LUMO sites are distinct in the active and low active compounds. In the active compound **1**, both HOMO and LUMO orbitals are overlapped significantly at A-ring and B-ring, while the carbonyl oxygen atom of amide and some positions of C-ring possess HOMO orbitals. Such extensive overlapping frontier orbitals indicated the highly reactive nature of the active compound. The HOMO orbital of **14** is located only at the carbonyl oxygen atom and C-ring. By comparing active **32** and low active **39**, the TZP core possesses both HOMO and LUMO orbitals, whereas B-ring and the linker between A-ring and B-ring have the obviously different electronic features. The computed quantum chemical descriptors HOMO and LUMO values of these four molecules in both gas and solvation (water) phases are summarized in Table S3 in the Supplementary Materials. For this analysis, because of the small values of both HOMO and LUMO energies (−0.24 to −0.21 eV and −0.08 to −0.04 eV), electron transfer and exchange may be equally possible, making these compounds very reactive. The HOMO-LUMO gap of the active compounds is slightly higher (~0.006 eV) when compared with that of low active compounds. Additionally, the dipole moments of these four compounds are also computed in both gas and solvation (water) phase (Table S3 in the Supplementary Materials). Higher values of dipole moments are obtained in the solvent phases (3.71 to 8.63 Debye) compared to the gas phase (3.39 to 6.09 Debye). This is due to the intermolecular interactions between the solvent and the studied compound. On the other hand, dipole moment represents a direct measure of the electron distribution in a molecule, which may give some insight on the degree of hydrophobicity/hydrophilicity of the compound. In particular, the two active compounds (**1** and **32**) show higher dipole moments than another two low active inhibitors (**14** and **39**), thus compounds **1** and **32** have much stronger hydrophobic interactions with the binding pocket than those of **14** and **39**.

### 3.5. Proposed Pharmacophore Model for 11β-HSD1 Inhibitors

**Figure 5:**
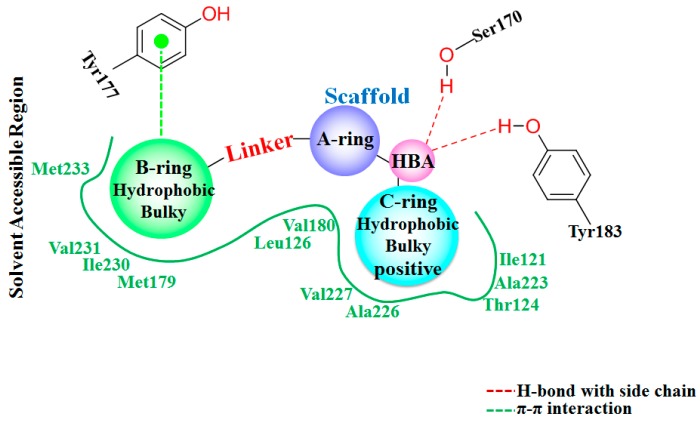
Summary of structure-activity relationships based on docking and 3D-QSAR investigation.

Combining the results from above docking simulations, 3D-QSAR analyses, and quantum chemistry computations, structural requirements for designing novel 11β-HSD1 inhibitors are summarized in Figure 5. A relatively conserved hydrogen bond accepter region (HBA) is essential for an inhibitor respecting the existence of residues Ser170 and Tyr183. Linker identity suggests that strict structural requirements at this position so that −SCH_2_− would be a suitable group. The B-ring was predicted to be surrounded by several hydrophobic residues, like Met179, Ile230, and Val231. It is consistent with the experimental activity that appropriate linker between A-ring and B-ring would also make the distal aromatic ring deep into this sub-region to interact with these hydrophobic residues. In addition, a moderately bulky group substituted on the 5′/6′-position of B-ring would increase the activity for the sake of the existence of Met233. Moreover, since the B-ring extends to a relatively capacious region at the surface of the protein, modification at specific position of B-ring may improve the physicochemical property of an inhibitor without sacrificing the potency. The bulky and hydrophobic group with positive charges potential of the C-ring are also predicted to be acceptable for the inhibitors. Thus, proper substitutions at these regions could directly contribute to improving the bioactivities of 11β-HSD1 inhibitors.

### 3.6. Molecular Dynamics Simulations

Although docking simulations provide a good starting for further calculations with the purpose of predicting the binding modes, the solvent effect on the whole system and the potential conformational changes are not fully taken into account. Therefore, molecular dynamics simulations were undertaken to investigate the stability of the enzyme-inhibitor complex in aqueous solution.

**Figure 6:**
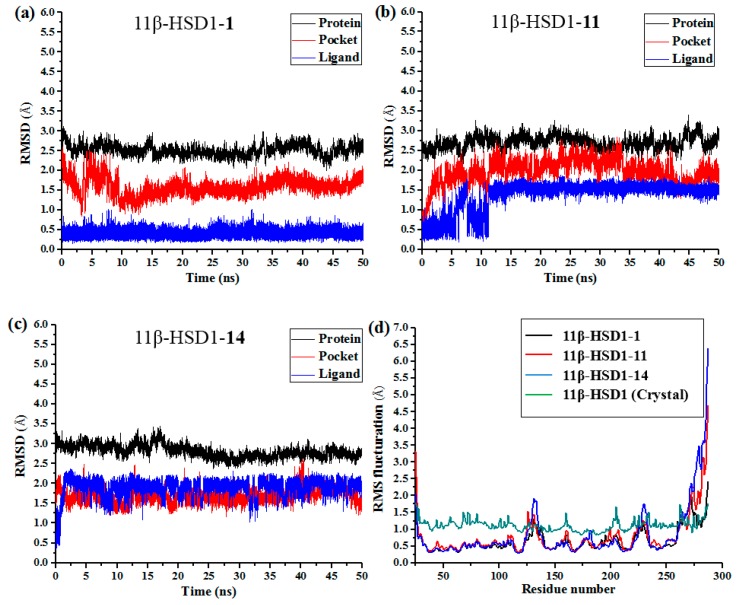
RMSDs of backbone atoms (C, Cα, and N) of the protein, backbone atoms of residues in the binding pocket (within 5 Å around each inhibitor), and the heavy atoms in the ligand for (**a**) 11β-HSD1-**1**; (**b**) 11β-HSD1-**11**; (**c**) 11β-HSD1-**14**; (**d**) RMSF of each residue of protein for all three complexes obtained from 50 ns MD simulations and crystal structure.

The docking-simulated structural models of compounds **1**, **11**, and **14** with 11β-HSD1, respectively, were applied as starting structures for MD simulations to analyze the structural requirements for the inhibitory activity and compare the binding modes between the inhibitors and 11β-HSD1. The root-mean-square deviations (RMSDs) for all heavy atoms of each ligand, backbone atoms of whole protein, and backbone atoms of residues in the binding pocket within 5 Å around the ligand were analyzed to explore the dynamic stabilities of all systems. The RMSDs of each system relative to their starting structures are plotted in Figure 6. As depicted in the plots, all three systems are in a relatively stable equilibrium in most of the time along the MD simulations procedures of 50 ns. Thus, it is reasonable to further calculate the binding free energies and free energy decomposition based on the conformations extracted from last 4 ns MD simulations trajectories. It was noticed that compounds **11** and **14** with lower biological activities spent longer time to reach equilibrium (Figure 6b,c). In addition, among all three complexes, **11** and **14** showed higher fluctuations than the most active compound **1**, with RMSD values of ~1.5 Å and ~2.0 Å for ligands, respectively, indicating that these two compounds would have some unstable binding mode in their complexes with 11β-HSD1.

In the meantime, the residue flexibility was also examined by analyzing the root mean square fluctuation (RMSF) of Cα atoms of protein in each complex. For comparison, the corresponding values of RMSF obtained from the co-crystal structure of 11β-HSD1-**1** are also shown in Figure 6d. The protein structures of these three systems with different inhibitors share similar RMSF distributions and trends of dynamics features. It shows that the residues with higher flexibilities distribute near the N- and C-terminal parts. The active site, including residues Ile121, Thr124, Leu126, Ser170-Ala172, Tyr177-Val180, Tyr183, Leu215-Ile218, Thr222-Ala223, Ile230-Val231, and Met233, exhibits rigid behavior. It also suggests that all inhibitors have similar interaction mechanism with 11β-HSD1. Accordingly, the observations were in agreement with the experimental X-ray crystallographic data, indicating the reliability of our MD results. By comparing the RMSF fluctuations of the binding pocket in the three complexes, it would be rational to conjecture that compound **1** has more stable interactions with 11β-HSD than the other two inhibitors, while **14** shows relatively higher structural mobility among the three complexes. Overall, these analyses of binding stabilization are consistent with the experimental activities.

### 3.7. Comparison of 3D-QSAR, Molecular Docking, and MD Simulation Results

**Figure 7:**
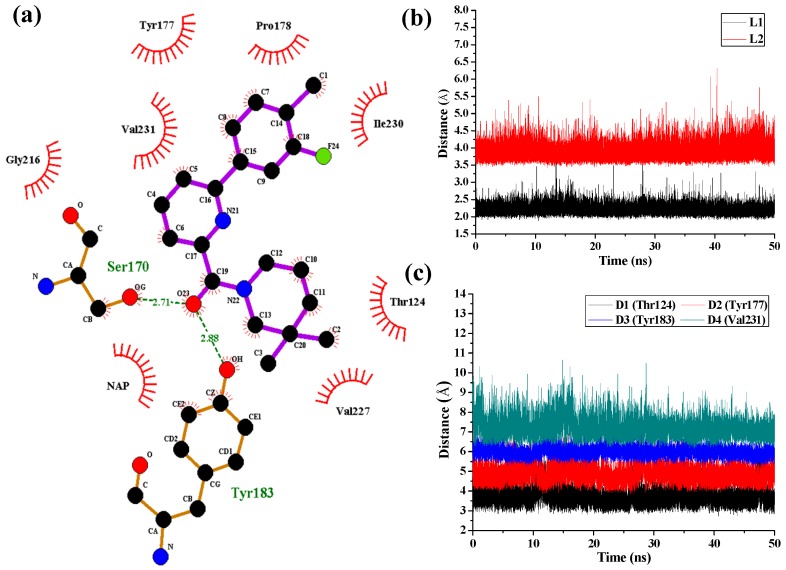
(**a**) 2D representation of H-bonds (Dashed lines) and hydrophobic interactions (spiked residues) with compound **1** generated by LIGPLOT program. (**b**) H-bond interactions of **1** in the binding site with time evolution. L1 and L2 represent the distances between the amide carbonyl oxygen atom and the hydroxyl groups of the side chains of Ser170 and Tyr183, respectively. The curves of L1 and L2 are shifted downward and upward by 0.5 Å and 1.0 Å, respectively. (**c**) Mass-center distances associated with hydrophobic interactions between 11β-HSD1 and **1** at the binding site over 50 ns of MD trajectories. The curve of D1 is shifted downward by 0.5 Å. D2, D3, and D4 are shifted upward by 1.0 Å, 2.0 Å, and 3.0 Å, respectively.

In order to further investigate the receptor-ligand interactions in the binding process, we compared the conformations of complexes during the MD simulations with the initial structures obtained from docking simulations. Clustering grouping was performed to analyze the structural variations of each complex during the MD simulations. Based on the pairwise similarity measured by RMSD with the *means* algorithm [57], five clusters were produced from the trajectory frames using the *ptraj* module of AMBER12. The representative conformations were then extracted from the cluster and compared with the above docking-simulated structures. In general, the conformation of the binding pocket and the inhibitors were found to be stable during the MD simulations, suggesting the rationality and validity of the docking models. However, some differences could still be observed between the MD simulated structures and the docked models. The program LIGPLOT [58] was used to generate 2D receptor-ligand interaction diagrams. Figure 7 shows the interaction features between the compound **1** and 11β-HSD1. 

The H-bond interactions with key residues in the active site along with H-bond length, angle and their occupancies for the three MD-simulated enzyme-inhibitor systems are listed in Table S4 in the Supplementary Materials. In the MD-simulated 11β-HSD1-**1** complex (Figure 7a), two stable H-bonds were formed for **1** to interact with adjacent residues around the binding pocket. One H-bond is formed between the carbonyl oxygen atom and the side chain hydroxyl group of Ser170 with an average distance of 2.71 Å and occupancy of 99.41%. Another one is formed between the carbonyl oxygen atom and the side chain phenolic hydroxyl group of Tyr183 with an average distance of 2.88 Å and occupancy of 99.43%. Figure 7b shows the H-bond lengths vs. time evolution. It can be seen that these two H-bonds were stable during MD simulations. In fact, above 3D-QSAR models showed a red contour in the electrostatic map of generated CoMFA model and a magenta region in the H-bond acceptor feature contours of generated CoMSIA model, suggesting that a highly electronegative atom is favorable for 11β-HSD1 bioactivity. Furthermore, we noticed that there were several hydrophobic contacts between the 3-fluoro-4-methylphenyl moiety and dimethylpiperidinyl moiety of compound **1** and residues Thr124, Tyr177, Pro178, Tyr183, Leu217, Val227, Ile230, and Val231, indicating that the hydrophobic interactions may also play critical roles in an inhibitor binding to 11β-HSD1. The interactions identified by MD-simulated models were consistent with the results from our generated steric contour maps in 3D-QSAR models, in which there were two green regions located around 5′-position of B-ring and 3′′-position of C-ring. Furthermore, our docking results indicated that the 3″,3″-dimethyl group of **1**′s C-ring remained into the hydrophobic region surrounded by the residues Ile121, Thr124, Val180, and Tyr183, and the space of the substituents on the B-ring are also limited by the residues Met179 and Val231. The stabilities of the hydrophobic interactions between 11β-HSD1 and **1** may be estimated by examining the time dependences of the related mass-center (CM) distances. As shown in Figure 7c, D1 and D3 represent the distances between the dimethylpiperidinyl ring of **1** and the main chains of Thr124 and Tyr183, respectively. D2 and D4 represent the distances between the 3-fluoro-4-methylphenyl moiety of **1** and the main chains of residues Tyr177 and Val231, respectively. The stability of the interatomic distances reveals that these hydrophobic interactions are also favorable to stabilize the binding of **1** to 11β-HSD1. Figure S5 in the Supplementary Materials describes the interaction mode between **11** and 11β-HSD1. As shown in Figure S5a, the amide carbonyl oxygen engaged in one H-bond with the critical residue Ser170 with an average distance of 2.69 Å and occupancy of 99.38%. It is worth noting that the residue Val231 has weaker hydrophobic interaction with **11** at the beginning (Figure S5c). This is due to the fact that the 2-chlorine phenyl of **11** has undergone several movements during the MD simulation. 

**Figure 8:**
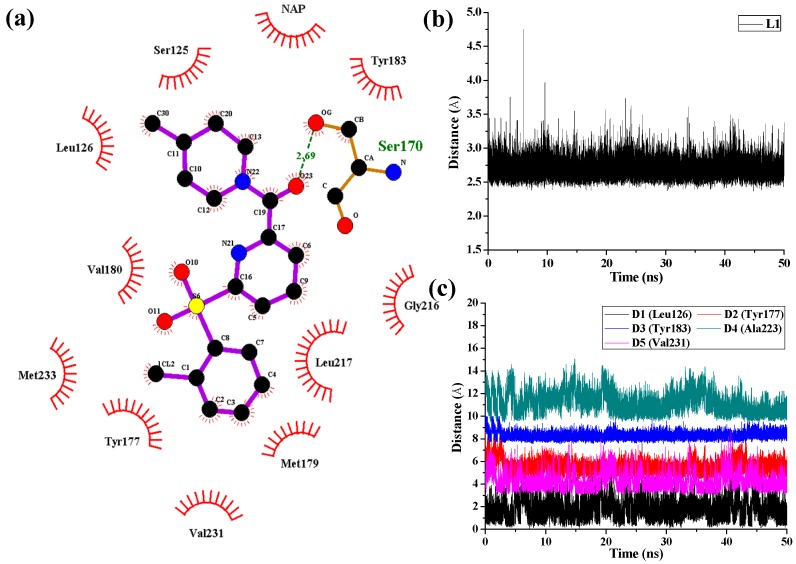
(**a**) H-bonds and hydrophobic interactions between 11β-HSD1 and compound **14** generated by LIGPLOT program; (**b**) H-bond interaction of **14** in the binding site with time evolution. L1 represents the distances between the amide carbonyl oxygen and the side chain hydroxyl of Ser170. (**c**) Mass-center distances associated with hydrophobic interactions between 11β-HSD1 and **14** in the binding site over 50 ns of MD trajectories. The curve of D1 is shifted downward by 3.0 Å. D2, D3, and D4 are shifted upward by 1.0 Å, 4.5 Å, and 6.0 Å, respectively.

As shown in Figure 8 in the case of 11β-HSD1-**14** complex, D1, D3, and D4 represent the distances between the 4-methylpiperidinyl ring of **14** and the main chains of Leu126, Tyr183, and Ala223, respectively. D2 and D5 represent the distances between the 2-chlorine phenyl moiety of **14** and the main chains of residues Tyr177 and Val231, respectively. As results, only one H-bond was found to be formed between the amide carbonyl oxygen and the side chain of Ser170 with an average distance of 2.69 Å and occupancy of 99.78%, while the hydrophobic interactions between **14** and its surrounding residues are relatively weaker. Moreover, the hydrogen bonding interactions between Tyr183 and compounds **11** and **14** were disappeared after MD simulation, indicating that the hydrogen bonding interactions between Tyr183 and these two inhibitors are relatively weak which led to decreases in their bioactivities. From the results discussed above, the ligands and the protein were both treated in a flexible way and the solvation effect was also taken into consideration during the MD simulations. However, the docking simulations ignored the flexibility of the protein when the ligands bound to the receptor. Therefore, MD simulations would not only confirm the rationality and effectiveness of the molecular docking and the 3D-QSAR models but also point out their defects in molecular modeling.

### 3.8. Binding Free Energy Calculation

The binding affinities of above three enzyme-inhibitors complexes were further calculated using the MM/PBSA method with the vibrational entropy term (*T*Δ*S*) estimated by the NMODE module of AMBER12. The results of estimated free energies and energy components of each complex in combination with their experimental data are listed in Table 2. Although the MM/PBSA-predicted binding free energies are moderately higher than the absolute experimental values (Δ*G*_exp_) determined from the corresponding experimental IC_50_ values, the ranking of predicted binding affinities (Δ*G*_pred(PB)_) of inhibitors **1** (−17.01 kcal/mol), **11** (−15.37 kcal/mol), and **14** (−10.26 kcal/mol) correlates well with their experimental ones (11β-HSD1 inhibitory potency of compounds **1**, IC_50_ = 0.1 nM, **11**, IC_50_ = 218 nM, and **14**, IC_50_ = 4670 nM). Therefore, MM/PBSA calculated results would be reliable for the analyses of the interaction mode of inhibitors binding to 11β-HSD1. In order to deeply understand which energy term has more impact on the binding of 11β-HSD1-inhibitor complexes, we compared four individual energy components, Δ*E*_ele_, Δ*E*_vdW_, Δ*G*_nonpol_, and Δ*G*_ele(PB)_ of these three complexes. As listed in Table 2, both the van der Waals interaction Δ*E*_vdW_ and the nonpolar solvation contribution Δ*G*_nonpol_ are of vital importance to the binding free energy. On the contrary, the sum (Δ*E*_ele_ +Δ*G*_ele(PB)_) of the electrostatic interaction and the polar solvation contribution is considerably unfavorable for binding free energy in all three complexes. Furthermore, it should be noted that Δ*E*_vdW_ is much stronger than Δ*G*_nonpol_, indicating that van der Waals interactions may contribute mostly to an inhibitor binding to 11β-HSD1 receptor. 

### 3.9. Free Energy Decomposition

**Figure 9:**
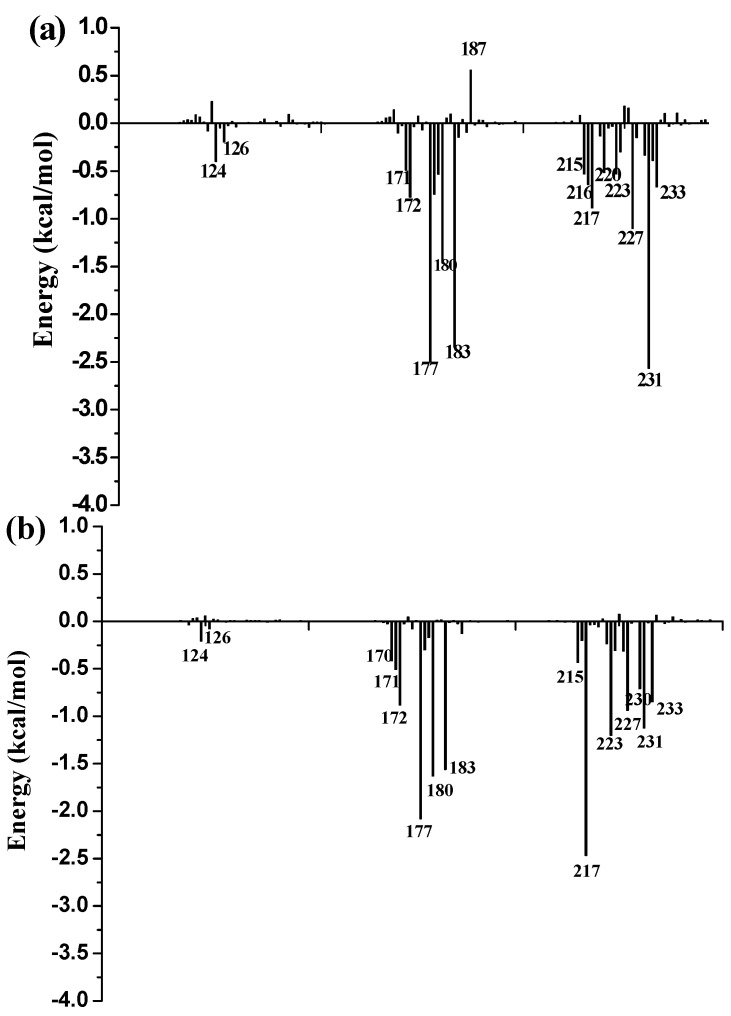

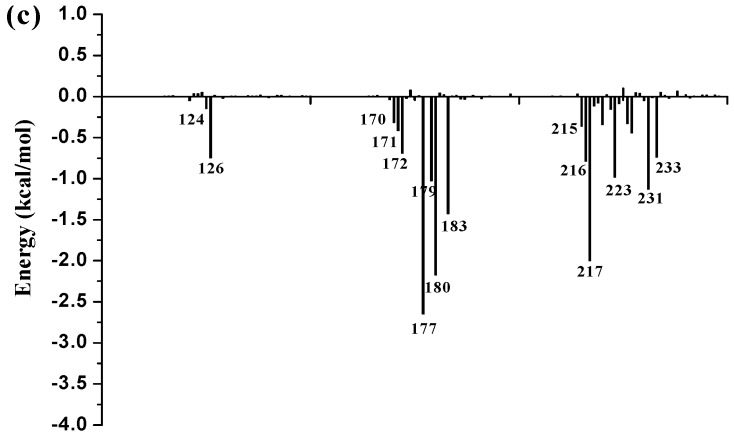
Per-residue binding free energy decomposition of: (**a**) 11β-HSD1-**1**; (**b**) 11β-HSD1-**11**; (**c**) 11β-HSD1-**14**.

To further identify key residues affecting the binding process, binding free energy was subsequently decomposed into the contribution of each residue by using the MM/GBSA method. The detailed interaction information of above three complexes is shown in Figure 9, and the energy contributions of the key residues for each inhibitor binding to 11β-HSD1 are compared in Figure S6 in the Supplementary Materials. As illustrated in Figure 9, major favorable energy contributions originate predominately from residues Leu171, Ala172, Tyr177, Val180, Tyr183, Leu215, Gly216, Leu217, Ala223, Val231, and Met233. 

Most of the key residues are hydrophobic enough to form strong van der Waals interactions with the inhibitors. Particular attention had been paid to those residues with relatively large differences in the contribution to binding free energies. Typically, the residues Ser170 and Tyr183, which have H-bond interactions with the inhibitor as discussed above, showed different interaction contributions. Ser170 has a little more energy contributions to **11** (−0.42 ± 0.02 kcal/mol) and **14** (−0.32 ± 0.02 kcal/mol) than **1** (−0.03 ± 0.02 kcal/mol), another residue Tyr183, which only has an H-bond interaction with compound **1** during MD simulations, has more energy contributions to **1** (−2.35 ± 0.02 kcal/mol) than **11** (−1.56 ± 0.02 kcal/mol) and **14** (−1.43 ± 0.02 kcal/mol). Among the resides in the binding pocket of 11β-HSD1, residues Thr124, Tyr183, Val227, and Val231 have stronger interactions with compound **1** in comparison with aforementioned residues, while residues Leu126, Met179, and Val180 interact with compound **14** differently from the other inhibitors. The residue Thr124 showed higher energy contributions to **1** (−0.40 ± 0.02 kcal/mol) binding to 11β-HSD1 in comparison with **11** (−0.21 ± 0.02 kcal/mol) and **14** (−0.05 ± 0.02 kcal/mol) since the side chain of Thr124 has interaction with the dimethylpiperidyl moiety of **1**. Among the hydrophobic residues in the binding pocket of 11β-HSD1, the residue Val231 contributes more than twice to interact with **1** (−2.57 ± 0.02 kcal/mol) than **11** (−1.12 ± 0.02 kcal/mol) and **14** (−1.13 ± 0.02 kcal/mol). This observation is consistent with the docking result that compound **1** with the 3-fluoro-4-methylphenyl moiety has strong hydrophobic interaction with the side chain of residue Val231. Therefore, we may safely conclude that Thr124, Tyr183, and Val 231 could be the key residues for inhibitors binding to 11β-HSD1. In order to gain insight into the energy contributions of the important residues mentioned above and to represent the results more intuitively, we divided each residue′s contribution into polar and nonpolar components (Figure S6 in the Supplementary Materials). As shown in Figure S6a, it is further confirmed that the van der Waals interactions and the nonpolar solvation energies regarding residues Thr124, Ala172, Tyr177, Pro178, Val227, and Val231 provide dominant forces for the binding process and are responsible for the difference between the binding free energies of the three complexes. As illustrated in Figure S6b, the total electrostatic and polar solvation energies of Ser170 and Val180 participate in yielding energetic differences for compound **14** from the other compounds. In general, Ser170 has significantly hydrophilic energy contributions for these three compounds, while Tyr183 has a favorable hydrophobic contribution to the binding of an inhibitor since its aromatic ring. In the meantime, Tyr183 also shows greater hydrophilic energy contributions for these three compounds. Therefore, Tyr183 plays a more critical role in forming H-bond interaction with an active inhibitor than Ser170. Taken together, we may conclude that the H-bond interactions with Ser170 and Tyr183, and the hydrophobic interactions with Thr124, Tyr177, Tyr183, Val227, and Val231 play important roles in improving the 11β-HSD1 inhibitory activity.

## 4. Conclusions

In the current study, a combined computational approach was applied to identify the structural determinants and specific binding modes between 11β-HSD1 and its inhibitors. The protein-ligand interactions have been characterized through receptor-based 3D-QSAR models, MESPs, MD simulations and free energy calculations. The higher statistic values for CoMFA and CoMSIA models demonstrated their reliability and predictive capability. From contour maps of the 3D-QSAR models, structure feature requirements were obtained for different substituents on the scaffold. It was worth noting that an important hydrogen bond rich region was identified by the contour maps. MESPs and Mulliken atomic charges analyses integrated with 3D-QSAR suggested that the electronegative amide carbonyl oxygen atom and nitrogen atoms of 1,2,4-triazolopyridine core in active compounds **1** and **32** were vital for the potency. Similarly, the molecular electrostatic profiles of the low active compounds **14** and **39** were also consistent with the 3D contour maps obtained from the CoMFA and CoMSIA models. Moreover, molecular dynamics simulations were performed to assess the contributions of residues to ligand binding by using three inhibitors **1**, **11**, and **14**, with different activities and diverse structure features. The calculated binding free energies were in good accordance with the biochemical results. The decomposition of binding free energy into each interaction components indicated that the van der Waals interactions provided the major driving force for the binding process, while free energy decomposition to each residue suggested that the residues Thr124, Ser170, Tyr177, Tyr183, Val227, and Val231 contributed predominately to the binding free energies. The pivotal hydrogen bond interactions with Ser170 and Tyr183 could help to enhance the inhibitory activity for 11β-HSD1. Overall, these results obtained from the computational approaches not only provide several possible mechanism interpretations at the molecular levels, but also can be helpful for the rational design of novel 11β-HSD1 inhibitors.

## Figures and Tables

**Table 1:** Statistical results of the CoMFA and CoMSIA models.

PLS Statistics	CoMFA	CoMSIA
*q*^2^ ^a^	0.656	0.523
ONC ^b^	5	6
*r*^2^ ^c^	0.964	0.936
SEE ^d^	0.286	0.390
*F* ^e^	132.964	58.058
Contribution		
Steric	0.586	0.104
Electrostatic	0.414	0.264
Hydrophobic		0.271
HB donor		0.180
HB acceptor		0.181
*r*^2^_pred_^f^	0.938	0.923

^a^ Leave-one out (LOO) cross-validated correlation coefficient; ^b^ Optimum number of principal components; ^c^ Non-cross-validated correlation coefficient; ^d^ Standard error of the estimate; ^e^ *F* value the Fischer ratio; ^f^ Correlation coefficient is derived from predictions of test set molecules.

**Table 2:** Binding free energy and its components for the studied complexes.

Protein-Inhibitor ^a^	11β-HSD1-1	11β-HSD1-11	11β-HSD1-14
Δ*E*_vdW_	−47.54	−44.56	−44.39
Δ*E*_ele_	−25.99	−18.19	−15.55
Δ*G*_nonpol_	−5.04	−4.67	−4.76
Δ*G*_ele(PB)_	41.07	36.00	35.85
Δ*E*_vdW_ + Δ*G*_nonpol_	−52.58	−49.24	−49.15
Δ*E*_ele_ + Δ*G*_ele(PB)_	15.08	17.81	20.30
*T*Δ*S*	−20.49	−16.06	−18.58
Δ*G*_prep(PB)_^b^	−17.01	−15.37	−10.26
IC_50_ (nM)	0.1	218	4670
Δ*G*_exp_^c^	−13.72	−9.14	−7.31

^a^ All energies are in kcal/mol. ^b^ Δ*G*_prep(PB)_: the calculated binding free energy by the MM/PBSA method. ^c^ Δ*G*_exp_: the experimental binding free energy calculated according to IC_50_ by Δ*G* ≈ −RT∙lnIC_50_.

## Supplementary Materials

Supplementary materials can be accessed at: http://www.mdpi.com/1420-3049/21/9/1222/s1.
