# Supplementary material for: Molecular Modeling Studies of 11β-Hydroxysteroid Dehydrogenase Type 1 Inhibitors through Receptor-Based 3D-QSAR and Molecular Dynamics Simulations
Source: Molecules. 2016 Sep 19;21(9):1222. doi: 10.3390/molecules21091222 (PMC6274164; doi:10.3390/molecules21091222)
Supplement: Supplementary file 1 [file molecules-21-01222-s001.pdf]

Supplementary Materials: Molecular Modeling Studies of 11β-Hydroxysteroid Dehydrogenase Type 1 Inhibitors Through Receptor-Based 3D-QSAR and Molecular Dynamics Simulations

Haiyan Qian, Jiongjiong Chen, Youlu Pan and Jianzhong Chen

Table S1. Structures and IC_{50} values of inhibitors used for 3D-QSAR modeling.

No.	Structure	Substituent		IC_{50} (nM)
		X	NR^2	
**1**	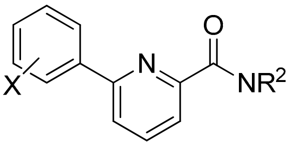	3-F, 4-Me	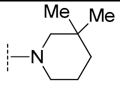	0.1
**2**		2-Cl	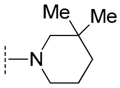	2.9
**3**		4-Cl	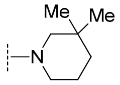	1.1
**4**		4-OCF_3	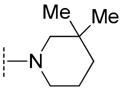	1.1
**5**		2-Cl	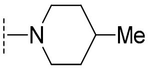	17
**6**		2-NO_2	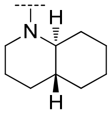	2.0
**7**		4-CF_3	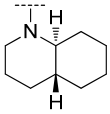	3.6
**8**		2-Ph	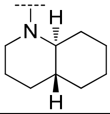	4.7
		L	X	
**9**	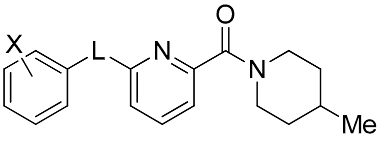	SCH_2	2,6-di-Cl	7.2
**10**		OCH_2	2,6-di-Cl	79
**11**		S	2-Cl	218
**12**		O	2-Cl	282
**13**		NH	2-Cl	381
**14**		SO_2	2-Cl	4670
		core		
**15**	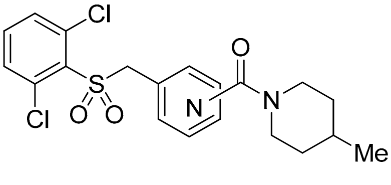	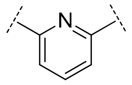		35
**16**		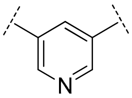		22
**17**		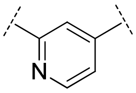		106
**18**		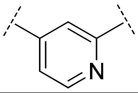		319

		**NR^2**		
**19**		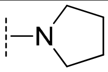		2140
**20**		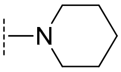		220
**21**	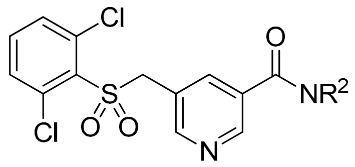	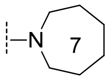		21
**22**		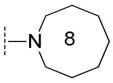		11
**23**		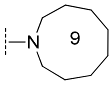		2.5
**24**		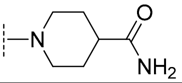		2350
		**Aryl**	**X-Y**	
**25**		2,6-diClPh	OCH_2	11
**26**		2,6-diClPh	CH_2O	578
**27**		2,6-diClPh	CH_2OCH_2	34
**28**	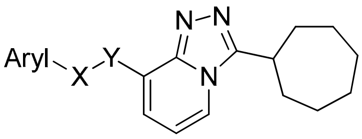	2,6-diClPh	O	36
**29**		2,6-diClPh	SO_2CH_2	2962
**30**		2,6-diClPh	SO_2NHCH_2	17
**31**		2,6-diClPh	SO_2NH	8778
**32**		2,6-diClPh	SCH_2	2.8
**33**		2-Me,3-ClPh	SO_2NHCH_2	12
		**R**		
**34**		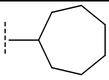		23
**35**		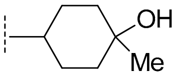		367
**36**		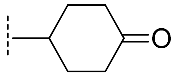		7804
**37**	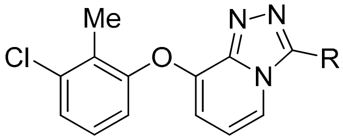	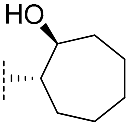		197
**38**		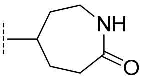		14030
**39**		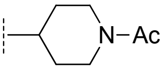		48240
**40** [a]		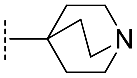		16060

[a] Compound **40** was used in non-protonated form.

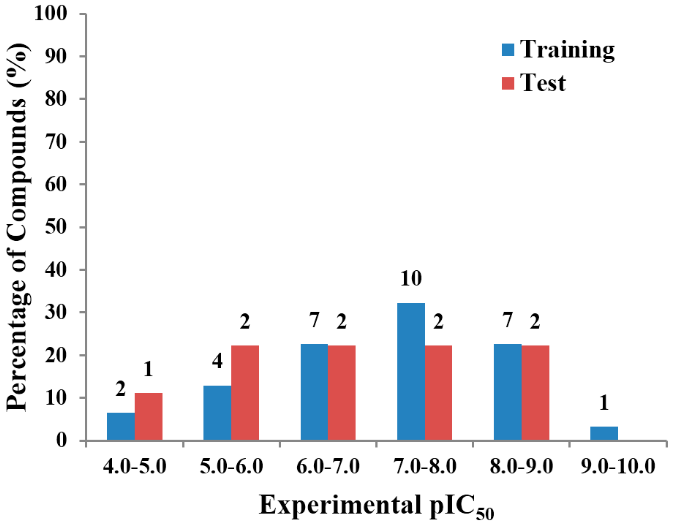

Figure S1. Bar graph shows the experimental inhibitory activities for training and test set compounds. The number of compound(s) in each interval is indicated above the column.

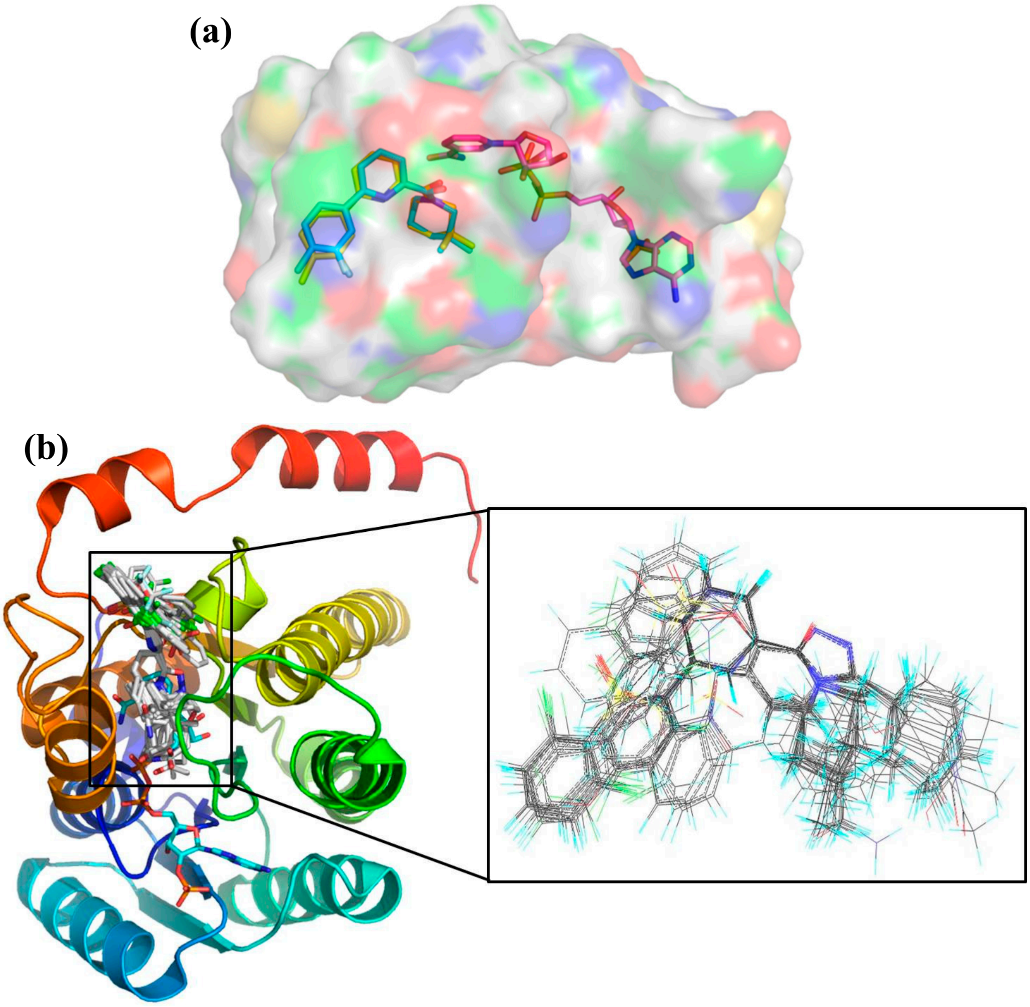

(c)

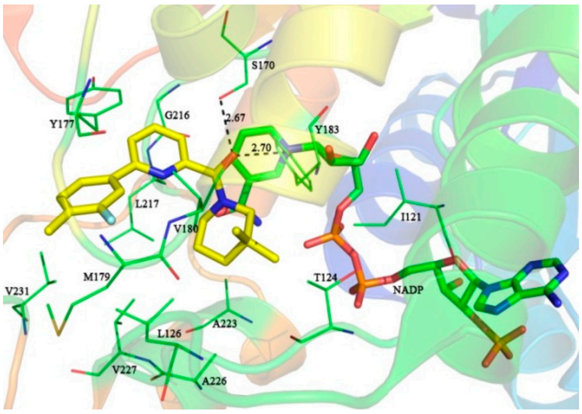

Figure S2. (**a**) The surface of the binding site and the conformational comparison of compound **1** from the docking result (blue) and co-crystallized ligand (yellow) in the 11β-HSD1 binding pocket. (**b**) Alignment results for the 40 examined compounds based on the docking conformations. (**c**) The detailed binding mode of compound **1** in 11β-HSD1.

(a)

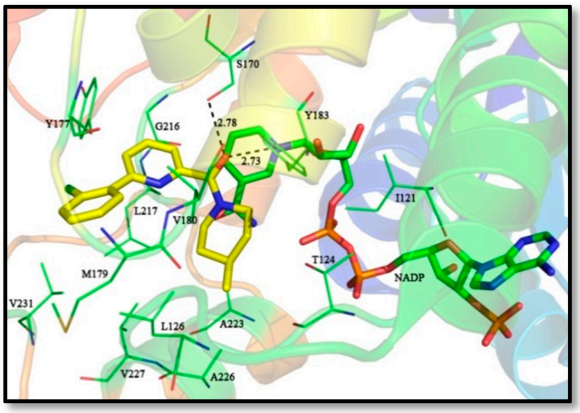

(b)

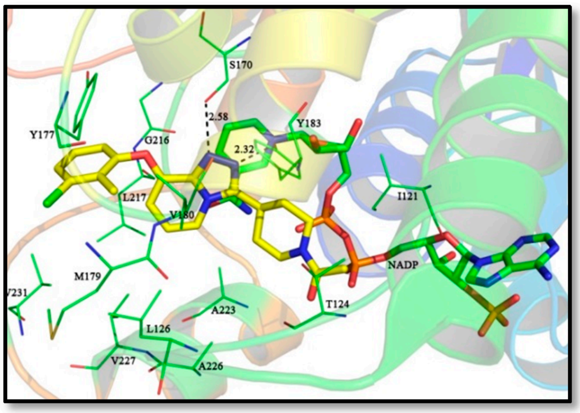

Figure S3. The detailed binding modes of complexes 11β-HSD1-**5** (**a**) and 11β-HSD1-**39** (**b**).

Table S2. Experimental and predicted pIC50 values and residuals of molecules in both training set and test set.

No.	pIC50					No.	pIC50				
	Exp.	CoMFA		CoMSIA			Exp.	CoMFA		CoMSIA	
		Pred.	Res.	Pred.	Res.			Pred.	Res.	Pred.	Res.
The training set of compounds						**26**	6.24	6.65	-0.41	6.48	-0.24
**1**	10.00	9.77	0.23	9.68	0.32	**27**	7.47	7.57	-0.10	7.56	-0.09
**2**	8.54	8.74	-0.2	8.68	-0.14	**29**	5.53	5.15	0.38	6.10	-0.57
**3**	8.96	8.98	-0.02	8.93	0.03	**30**	7.77	7.64	0.13	8.05	-0.28
**4**	8.96	9.11	-0.15	9.33	-0.37	**31**	5.06	5.08	-0.02	5.25	-0.19
**5**	7.77	7.69	0.08	8.01	-0.24	**33**	7.92	7.97	-0.05	8.27	-0.35
**7**	8.44	8.22	0.22	8.53	-0.09	**35**	6.44	6.29	0.15	6.66	-0.22
**8**	8.33	8.33	0.00	8.53	-0.20	**36**	5.11	4.99	0.12	4.97	0.14
**9**	8.14	7.89	0.25	7.90	0.24	**37**	6.71	6.69	0.02	6.72	-0.01
**10**	7.10	7.18	-0.08	7.60	-0.50	**38**	4.85	4.74	0.11	5.06	-0.21
**12**	6.55	6.36	0.19	6.60	-0.05	**39**	4.32	4.66	-0.34	3.74	0.58
**13**	6.42	6.62	-0.20	6.98	-0.56	The test set of compounds					
**14**	5.33	5.68	-0.35	4.89	0.44	**6**	8.70	8.88	-0.18	8.70	0.00
**15**	7.46	7.47	-0.01	7.43	0.03	**11**	6.66	6.26	0.40	6.46	0.20
**16**	7.66	7.65	0.01	7.15	0.51	**18**	6.50	6.49	0.01	6.65	-0.15
**17**	6.97	7.18	-0.21	7.03	-0.06	**19**	5.67	5.38	0.29	6.38	-0.71
**20**	6.66	6.86	-0.20	6.98	-0.32	**24**	5.63	5.59	0.04	5.76	-0.13
**21**	7.68	7.77	-0.09	7.47	0.21	**28**	7.44	7.81	-0.37	6.96	0.48
**22**	7.96	8.06	-0.10	7.82	0.14	**32**	8.55	8.51	0.04	8.07	0.48
**23**	8.60	8.53	0.07	7.75	0.85	**34**	7.64	7.05	0.59	7.08	0.56
**25**	7.96	7.78	0.18	7.71	0.25	**40**	4.79	5.21	-0.42	4.93	-0.14

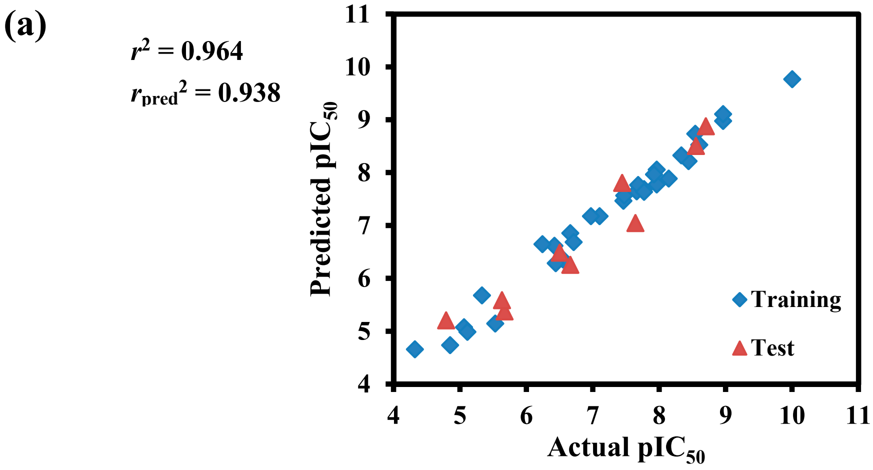

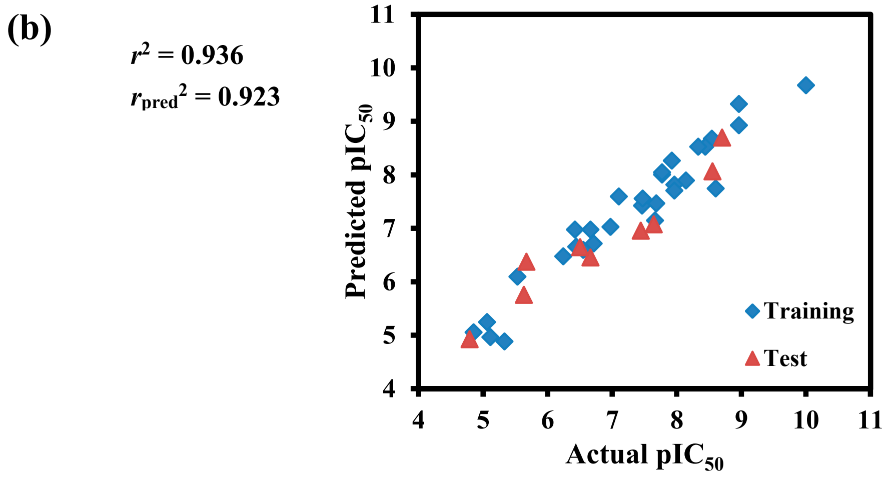

Figure S4. (**a**) Plots of predicted versus experimental pIC50 values of training (blue) and test (red) set of the CoMFA model. (**b**) Plots of predicted versus experimental pIC50 values of training (blue) and test (red) set of the CoMSIA model.

Table S3. Calculated quantum chemical descriptors for the active compounds (**1** and **32**) and low active compounds (**14** and **39**).

Comp No.	Gas Phase				Comp No.	Solvent Phase (Aqueous)			
	HOMO (eV)	LUMO (eV)	HLG (eV)	Dipole (D)		HOMO (eV)	LUMO (eV)	HLG (eV)	Dipole (D)
**1**	−0.224	−0.043	0.181	3.858	**1**	−0.231	−0.048	0.183	5.342
**32**	−0.213	−0.054	0.159	6.091	**32**	−0.217	−0.055	0.162	8.625
**14**	−0.237	−0.079	0.158	3.394	**14**	−0.237	−0.077	0.160	4.422
**39**	−0.217	−0.042	0.175	3.494	**39**	−0.216	−0.039	0.177	3.711

Table S4. Hydrogen bonds analyses from MD simulations [a].

System	Donor	Acceptor	Occupancy (%) [b]	Distance (Å) [c]	Angle (°) [d]
11β-HSD1-**1**	Ser170 HG	ligand O23	99.41	2.71 (0.12)	20.48 (10.79)
	Tyr183 HH	ligand O23	99.43	2.88 (0.21)	21.51 (11.57)
11β-HSD1-**11**	Ser170 HG	ligand O23	99.38	2.69 (0.12)	25.17 (11.12)
11β-HSD1-**14**	Ser170 HG	ligand O23	99.78	2.69 (0.12)	22.81 (10.30)

[a] The listed donor and acceptor pairs satisfy the criteria (H-bond length less than 5 Å and H-bond angle in the range of 120-180°) for the H-bond over 30.0% of the time during the 50 ns of simulation. [b] Occupancy is in unit of percentage of H-bond formed during the investigated time period. [c] The average distance with standard error (SE = standard deviation/$N^{1/2}$) in parentheses between H-bond acceptor atom and H-bond donor atom in the investigated time period. [d] The average angle with standard error (SE = standard deviation/$N^{1/2}$) in parentheses for H-bond in the investigated time period.

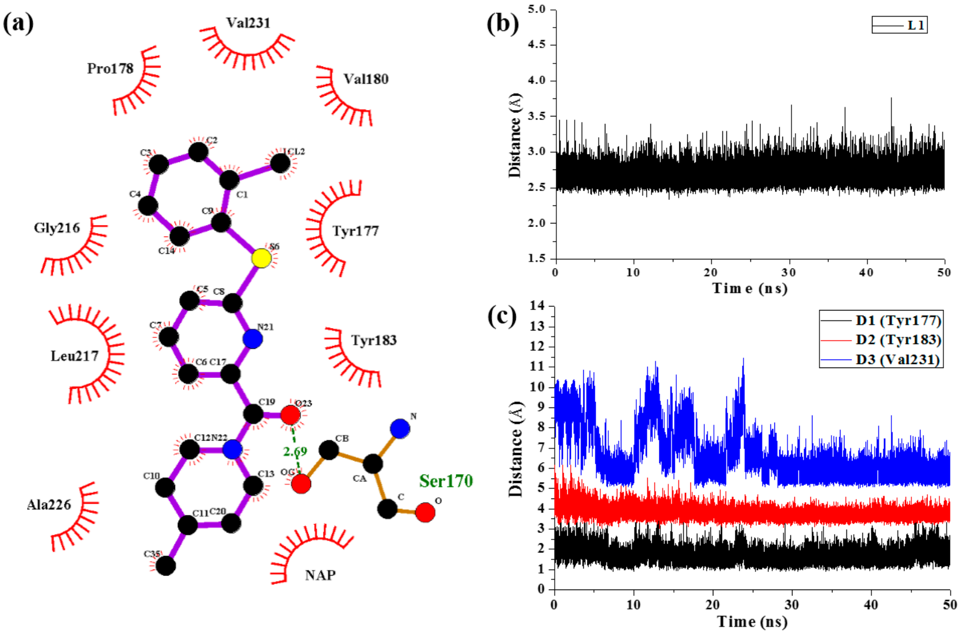

Figure S5. (**a**) H-bonds and hydrophobic interactions between 11β-HSD1 and compound **11** generated by LIGPLOT program. (**b**) H-bond interaction of **11** in the binding site with time evolution. L1 represents the distances between the amide carbonyl oxygen and the side chain hydroxyl of Ser170. (**c**) Mass-center distances associated with hydrophobic interactions between 11β-HSD1 and **11** at the binding site over 50 ns of MD trajectories. The curves of D1 is shifted downward by 2.0 Å, D3 is shifted upward by 2.0 Å, respectively.

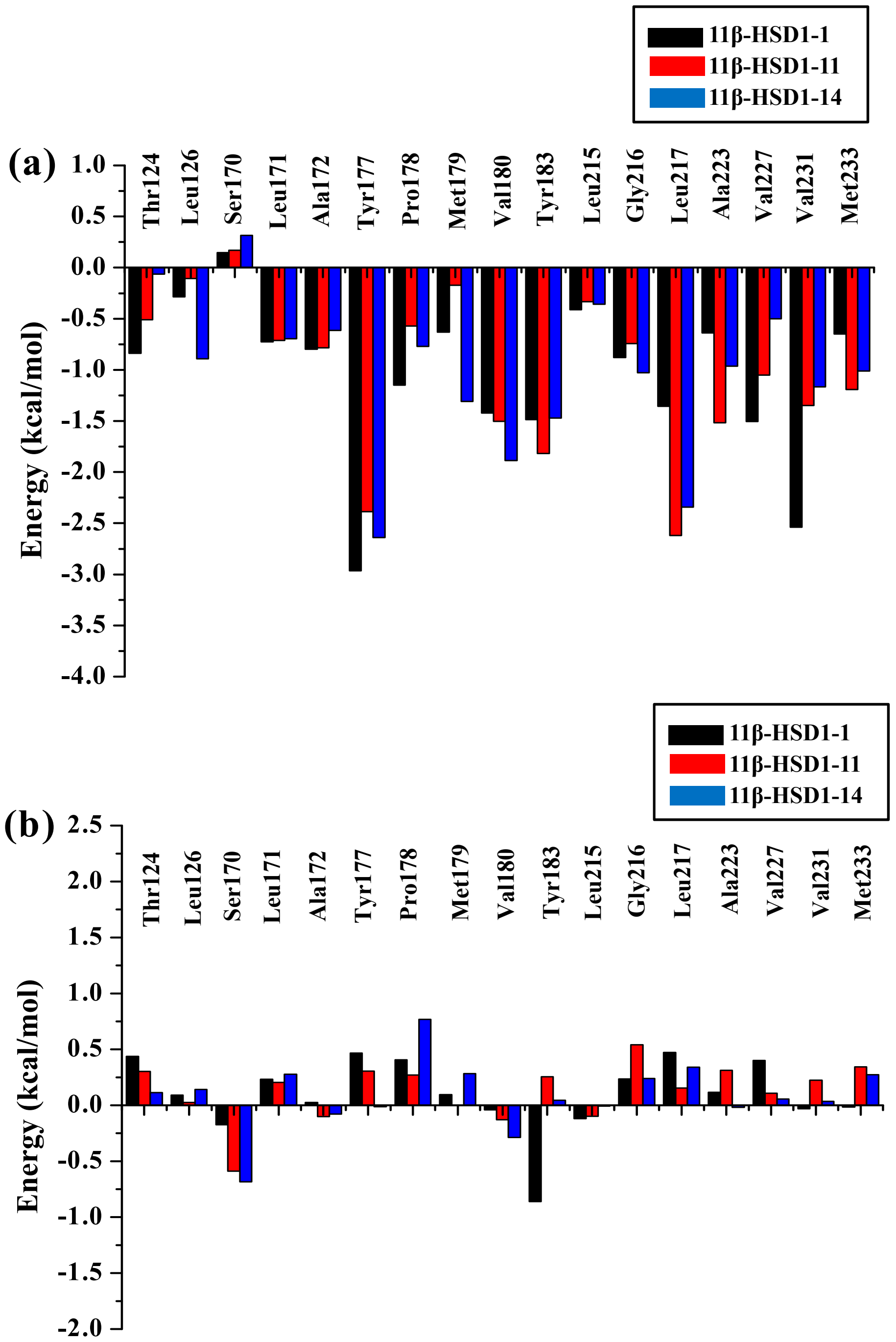

Figure S6. Comparison of per-residue energy decomposition for key residues for the three enzyme-inhibitors complexes: (**a**) the sum of vdW and nonpolar solvation energy, $\Delta E_{vdW} + \Delta G_{nonpol}$, and (**b**) the sum of electrostatic and polar solvation energy, $\Delta E_{ele} + \Delta G_{ele(PB)}$.
